# Supplementary material for: Cross-catenation between position-isomeric metallacages
Source: Nat Commun. 2024 Feb 14;15:1363. doi: 10.1038/s41467-024-45681-6 (PMC10866959; doi:10.1038/s41467-024-45681-6)
Supplement: Supplementary file 1 — Supplementary Information [file 41467_2024_45681_MOESM1_ESM.pdf]

Supplementary Information

Cross-catenation between two position-isomeric metallacages

Wang et al.

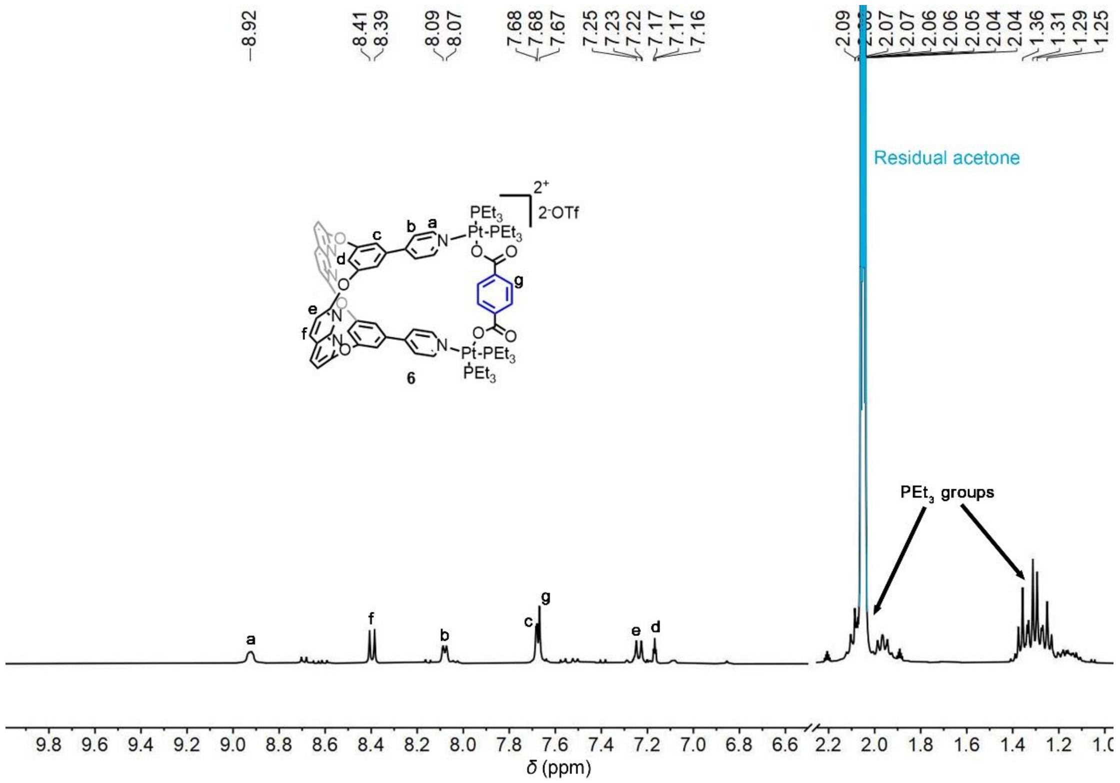

Supplementary Figure 1. ^{1}H NMR spectrum (400 MHz, acetone-d_6, 298K) of **6** (1 mM).

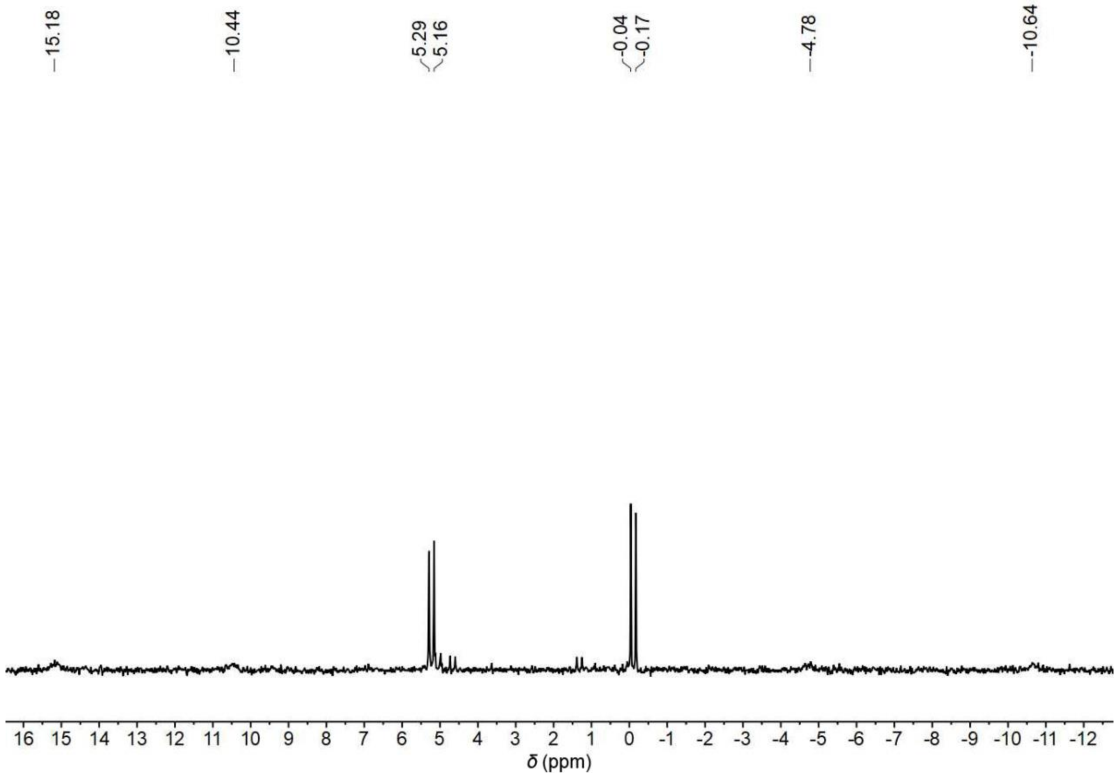

Supplementary Figure 2. ^{31}P{^{1}H} NMR spectrum (160 MHz, acetone-d_6, 298K) of **6** (1 mM).

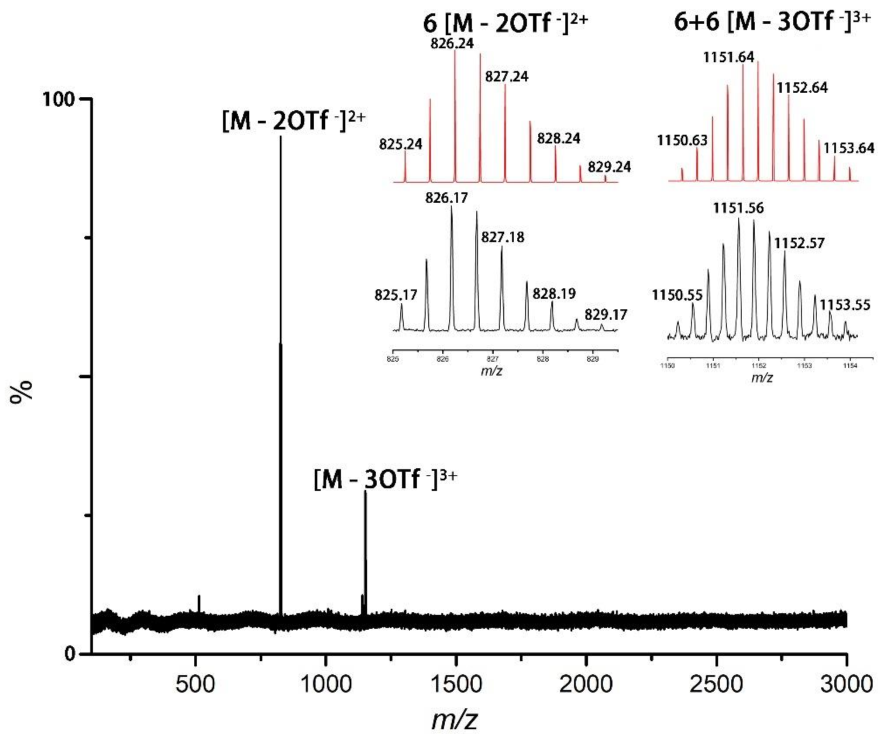

Supplementary Figure 3. Experimental (black) and calculated (red) electrospray ionization mass spectrum of **6** ([M −2OTf$^-$]$^{2+}$) and [2]catenane **6+6** ([M – 3OTf$^-$]$^{3+}$).

To be noticed, mass signal of [2]catenane **6+6** was also observed (1151.56, [M – 3Otf$^-$]$^{3+}$), suggesting that catenated structures are co-existed in the solution.

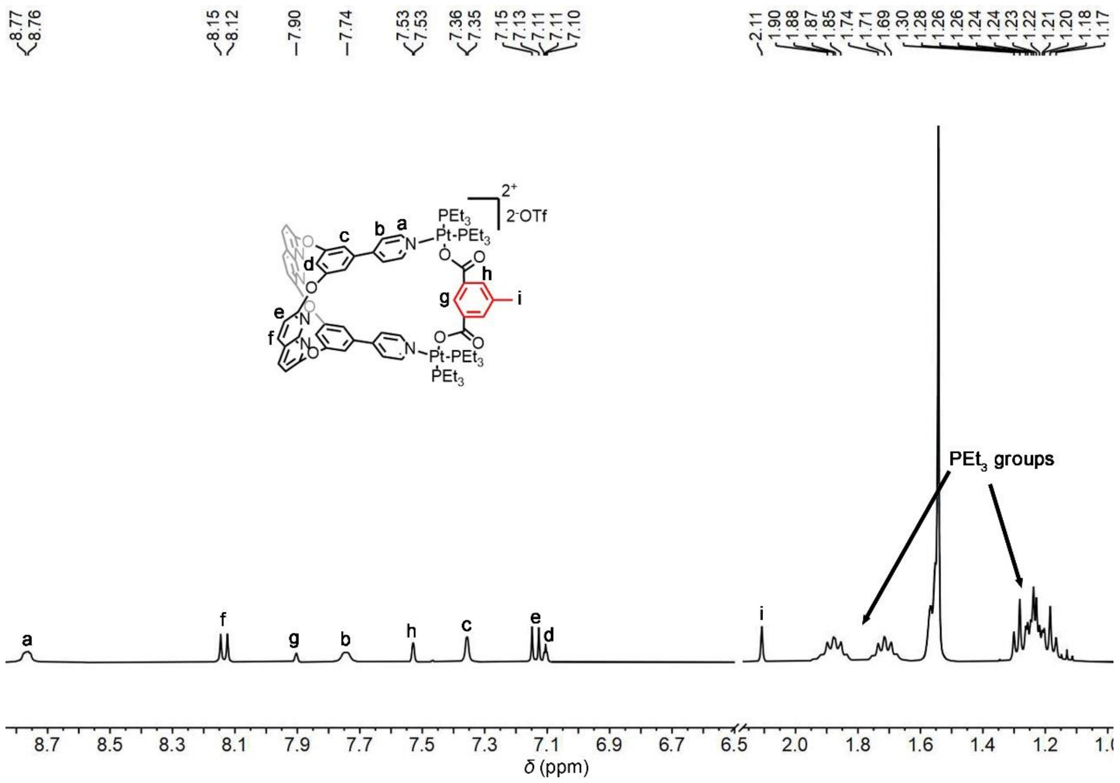

Supplementary Figure 4. ^{1}H NMR spectrum (400 MHz, CD_2Cl_2, 298K) of **8**.

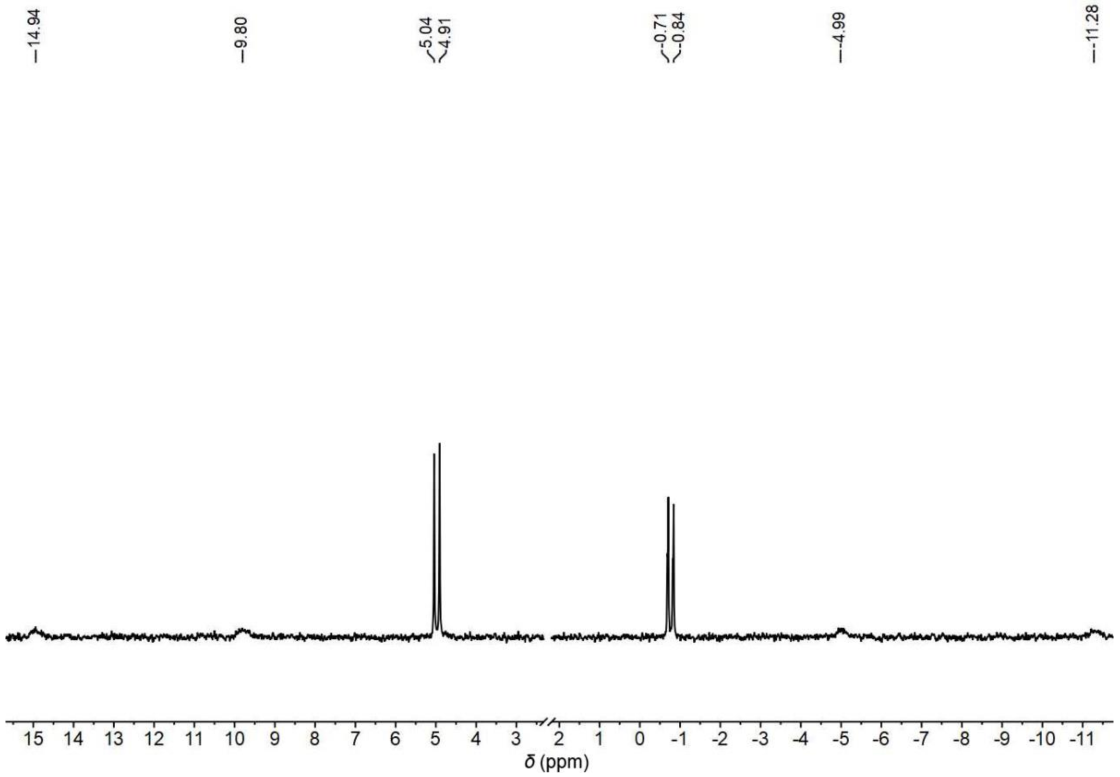

Supplementary Figure 5. $^{31}P\{^{1}H\}$ NMR spectrum (160 MHz, acetone-d_6, 298K) of **8** (1 mM).

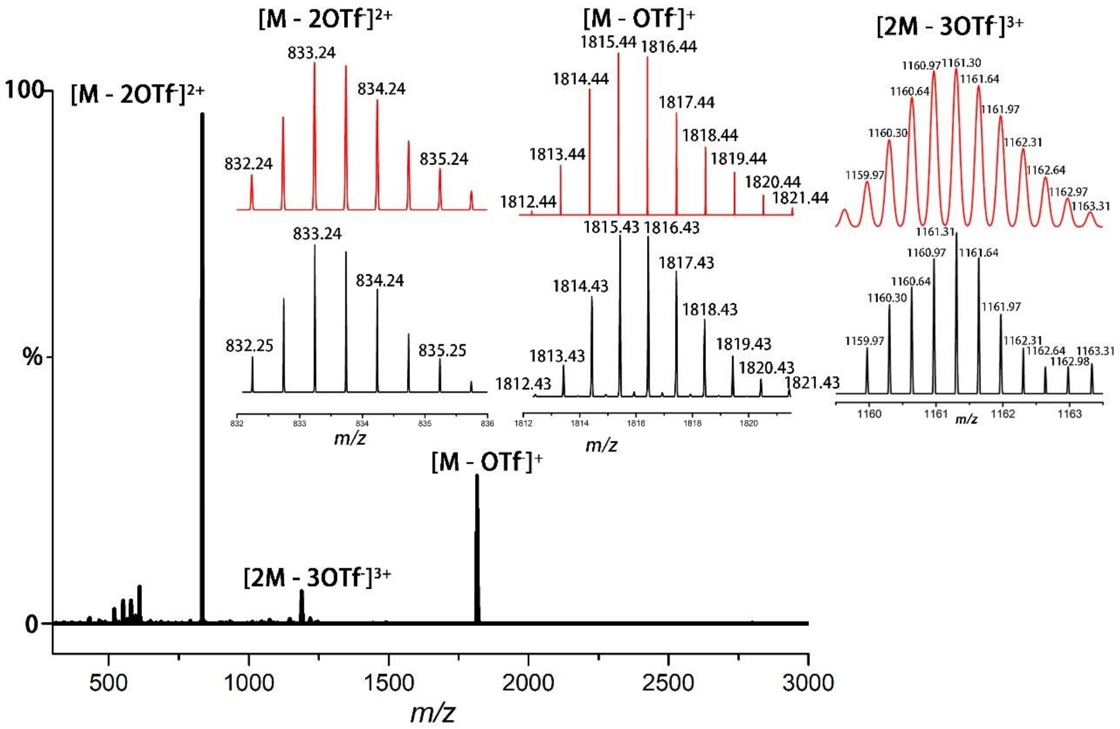

Supplementary Figure 6. Experimental (black) and calculated (red) electrospray ionization mass spectrum of **8** ([M −OTf⁻]⁺ & [M −2OTf⁻]²⁺) and **8+8** ([2M-3OTf⁻]³⁺)

Supplementary Table 1. Containing the Check cif alerts obtained from the checkcif.iucr.org webpage and the authors responses

Check cif alerts	Response
The value of sine(theta_max)/wavelength is less than 0.550	The diffraction limit was found and the data was trimmed accordingly.
Poor Data / Parameter Ratio	The diffraction limit was found due to the qualities of crystal.
Atom has ADP max/min Ratio	Disordered moieties remained unmodelled due to the low data/parameter ratio.
Large Average Ueq of Residue	Disordered moieties remained

Including	unmodelled due to the low data/parameter
Singly Bonded Carbon Detected (H-atoms Missing).	Hydrogen atoms were not placed in some PEt_3 groups' calculated positions to avoid disorder.
Low Bond Precision on C-C Bonds	Disordered moieties remained unmodelled due to the low data/parameter ratio.
Ratio Observed / Unique Reflections (too) Low	The diffraction limit was found due to the qualities of crystal.
High wR2 Value (i.e. > 0.25)	Disordered moieties remained unmodelled due to the low data/parameter.
High 'MainMol' Ueq as Compared to Neighbors of	Disordered moieties remained unmodelled due to the low data/parameter

Supplementary Table 2. Crystal data and structure refinement for **6** & **6+6**

Empirical formula	$C_{72}H_{82}F_6N_6O_{14}P_4Pt_2S_2$
Formula weight	1947.61
Temperature	103.15 K
Wavelength	0.71073 Å
Crystal system	triclinic
Space group	P -1
Unit cell dimensions	a = 18.645(3) Å α = 73.533(3)°.
	b = 18.959(3) Å β = 71.591(3)°.
	c = 28.394(4) Å γ = 65.526(3)°.
Volume	8531(2) Å^3
Z	4

Density (calculated)	1.516 Mg/m^3
Absorption coefficient	3.473 mm^{-1}
F(000)	3880
Crystal size	0.152 x 0.12 x 0.006 mm^3
Theta range for data collection	1.295 to 19.397°.
Index ranges	-17<=h<=17, -17<=k<=17,
	-26<=l<=26
Reflections collected	82310
Independent reflections	14543 [R(int) = 0.0835]
Refinement method	Full-matrix least-squares on F^2
Data / restraints / parameters	14543 / 2256 / 1933
Goodness-of-fit on F^2	0.916
Final *R* indices [*I*>2sigma(*I*)]	R_1 = 0.0651, wR_2 = 0.1535
R indices (all data)	R_1 = 0.1121, wR_2 = 0.1723
Extinction coefficient	0.00013(5)
Largest diff. peak and hole	1.199 and -1.195 e.Å^{-3}
CCDC	2178656

Supplementary Table 3. Crystal data and structure refinement for **4+6**

Empirical formula	$C_{69.87}H_{26}N_6O_{7.78}P_4Pt_2$
Formula weight	1587.98
Temperature	193 K
Wavelength	0.71073 Å
Crystal system	triclinic
Space group	P -1
Unit cell dimensions	*a* = 18.21(4) Å *α* = 73.43(11)°.
	b = 19.53(5) Å *β* = 72.40(9)°.
	c = 28.81(5) Å *γ* = 65.34(6)°.
Volume	8723(35) Å^3

Z	4
Density (calculated)	1.209 Mg/m^3
Absorption coefficient	3.473 mm^{-1}
F(000)	3062
Crystal size	0.52 x 0.13 x 0.006 mm^3
Theta range for data collection	1.167 to 24.999^o.
Index ranges	-21<=h<=21, -23<=k<=22,
	-34<=l<=34
Reflections collected	81835
Independent reflections	30246 [R(int) = 0.0807]
Refinement method	Full-matrix least-squares on F^2
Data / restraints / parameters	30246 / 3315 / 1630
Goodness-of-fit on F^2	0.906
Final R indices [I>2sigma(I)]	R_1 = 0.0983, wR_2 = 0.2871
R indices (all data)	R_1 = 0.1981, wR_2 = 0.3589
Extinction coefficient	0.00073(11)
Largest diff. peak and hole	1.308 and -0.870 e.Å^{-3}
CCDC	2178655

Crystal Structural figures:

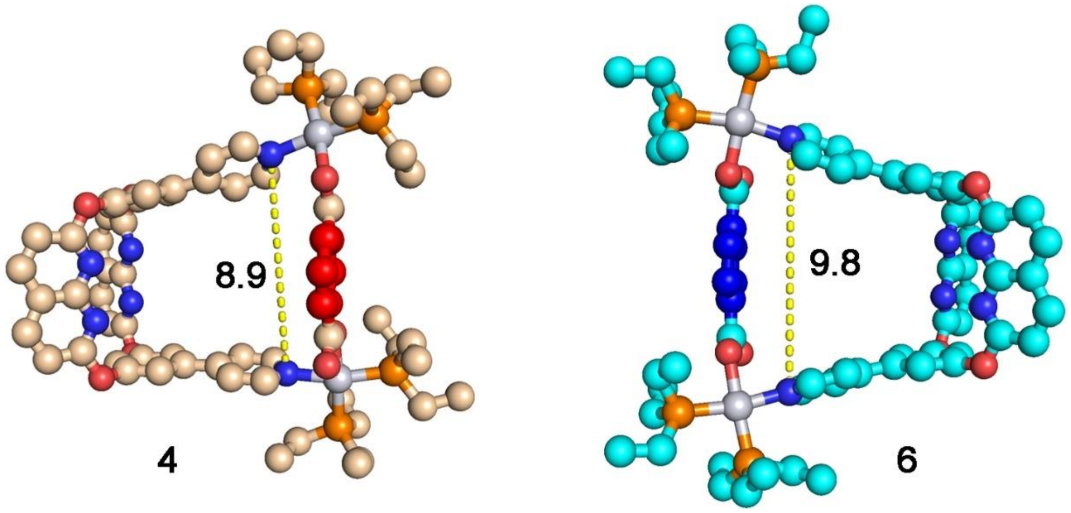

Supplementary Figure 7. Crystal structures of metallacages **4** and **6** (distances in Å; hydrogen atoms omitted for clarity).

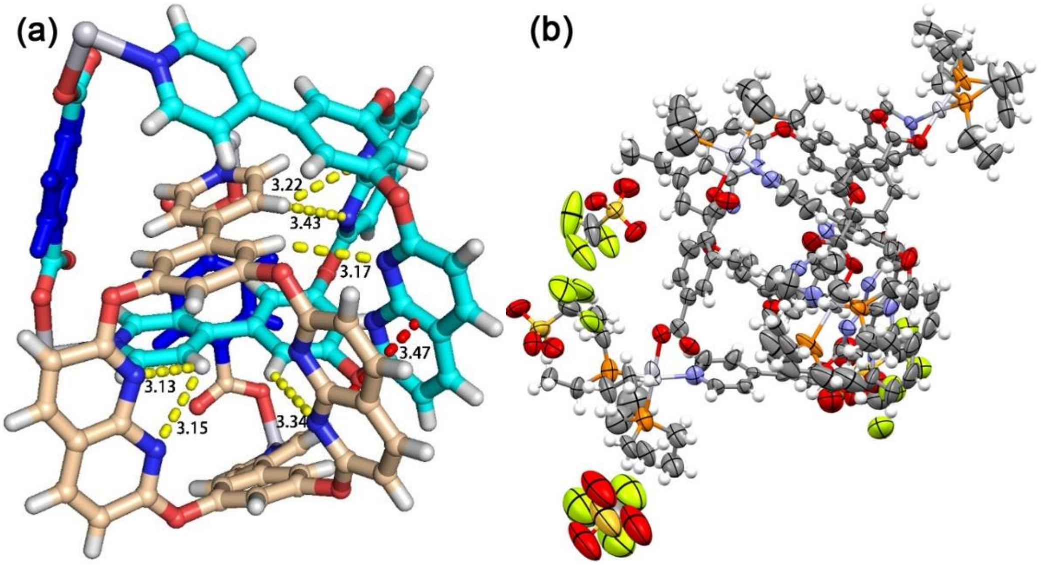

Supplementary Figure 8. (a) Multiple C-H···N hydrogen bonds between H atoms from aromatic rings and N atoms from naphthyridyl groups of **6+6** (yellow lines) and π···π stacking (red line) between two of four naphthyridine rings of **6+6** in solid state: C-H···N distances (Å); centroid···centroid distance (Å). (PEt_3 groups are omitted) (b) ORTEP-style crystal structure of [2]catenane **6+6**.

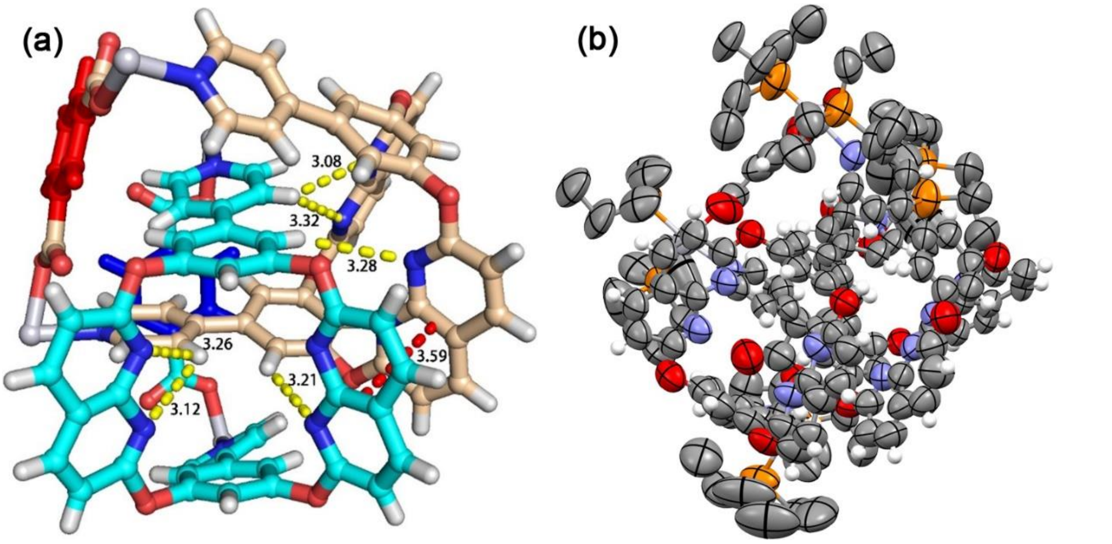

Supplementary Figure 9. Multiple C-H···N hydrogen bonds between H atoms from aromatic rings and N atoms from naphthyridyl groups of **4+6** (yellow lines) and π···π stacking (red line) between two of four naphthyridine rings of **4+6** in solid state: C-H···N distances (Å); centroid···centroid distance (Å). (PEt_3 groups are omitted) (b) ORTEP-style crystal structure of cross-catenane **4+6**.

Individual Concentration dependent NMR experiments

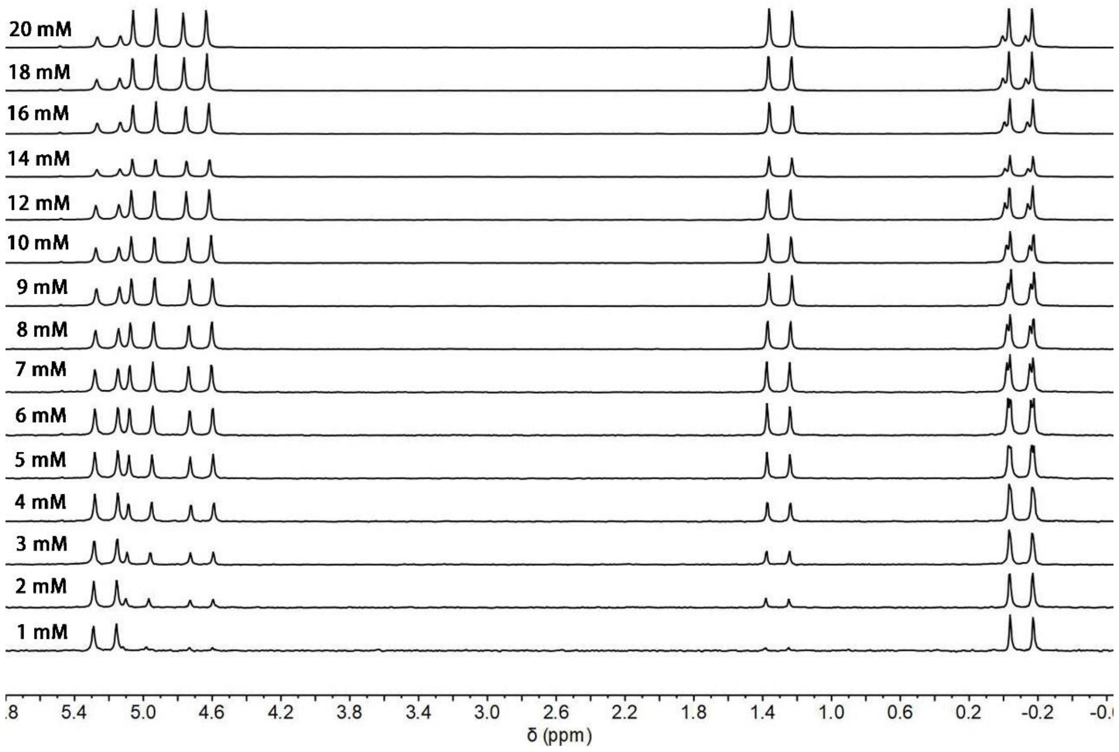

Supplementary Figure 10. $^{31}P\{^{1}H\}$ NMR (160 MHz, acetone-d_6, 298K) spectra of **6** at different concentrations.

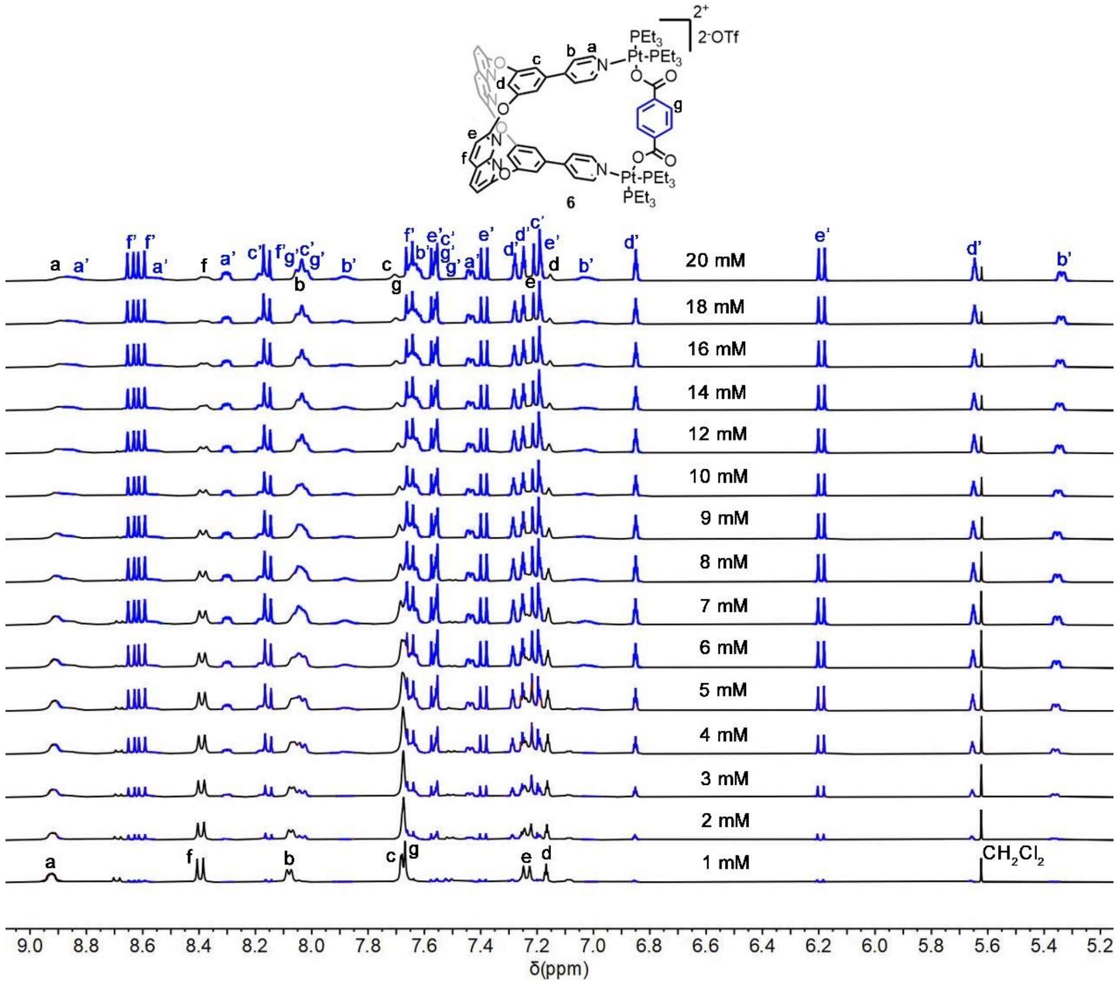

Supplementary Figure 11. ^{1}H NMR (400 MHz, acetone-d_6, 298K) spectra of **6** at different concentrations.

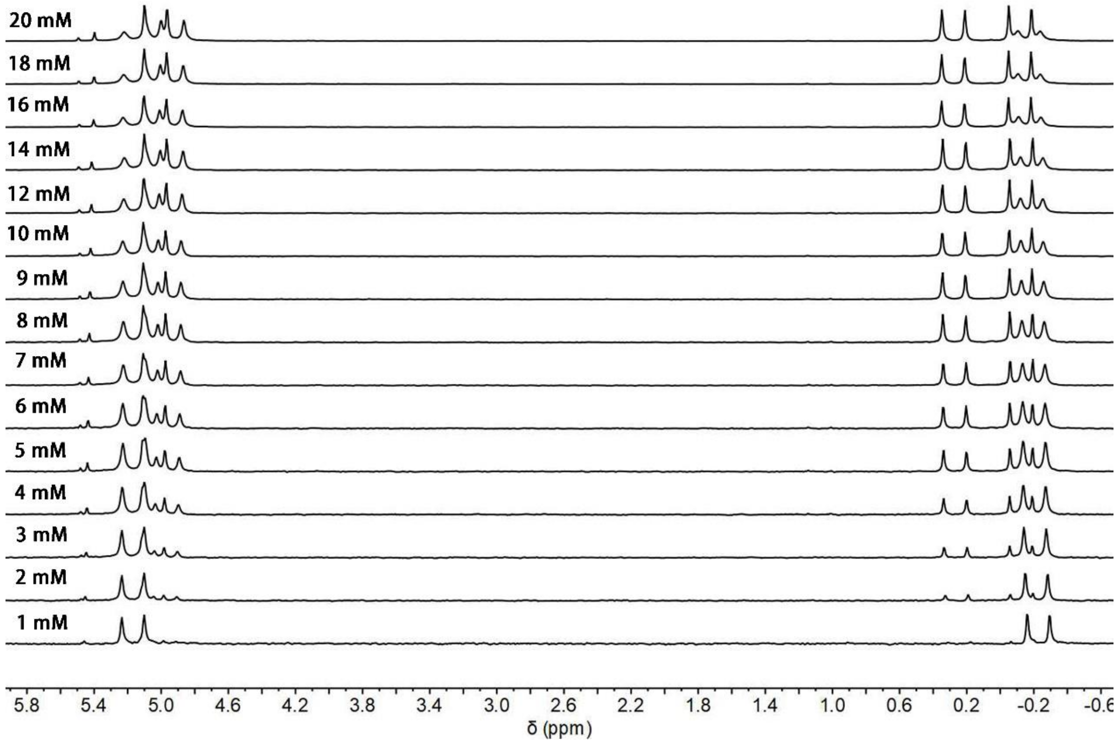

Supplementary Figure 12. $^{31}P\{^{1}H\}$ NMR (160 MHz, acetone-d_6, 298K) spectra of **4** at different concentrations.

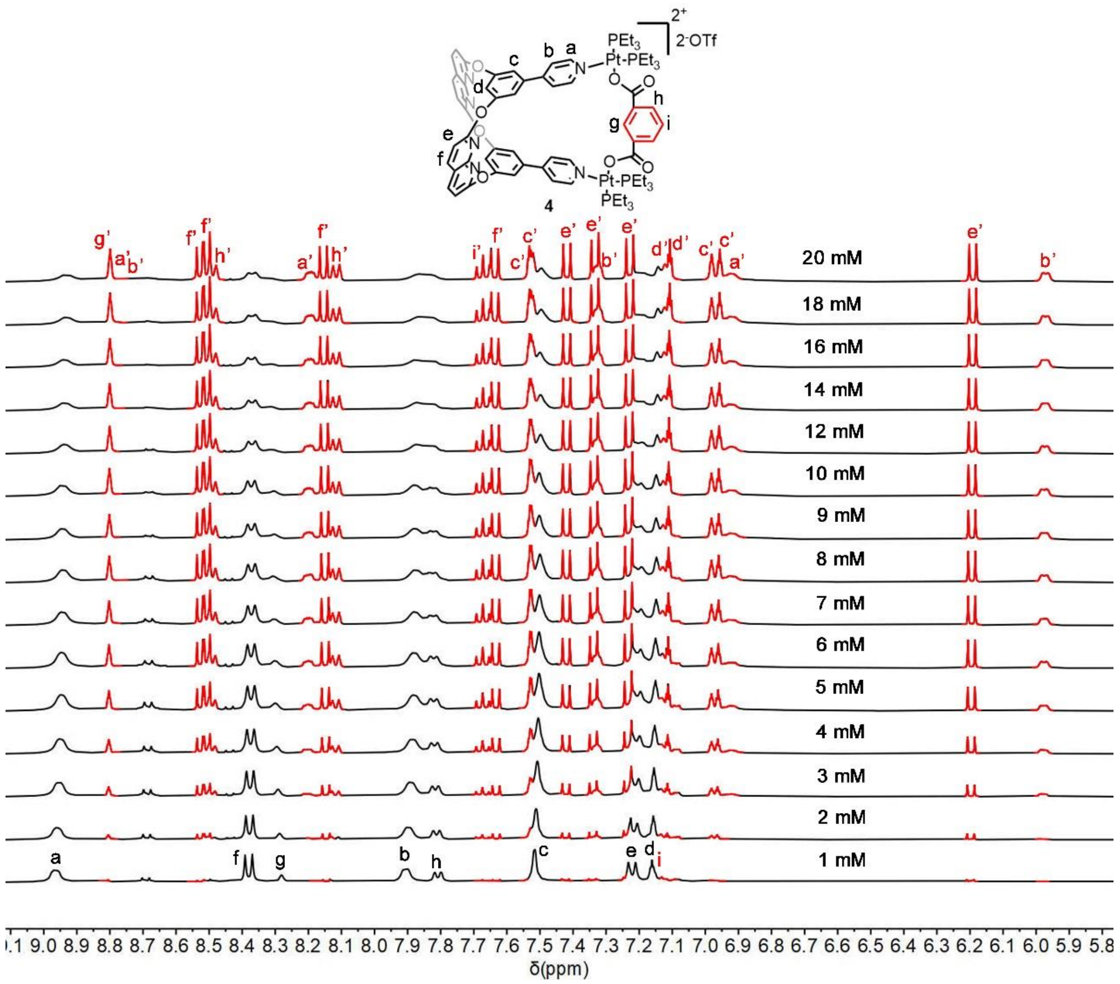

Supplementary Figure 13. ^{1}H NMR (400 MHz, acetone-d_6, 298K) spectra of **4** at different concentrations.

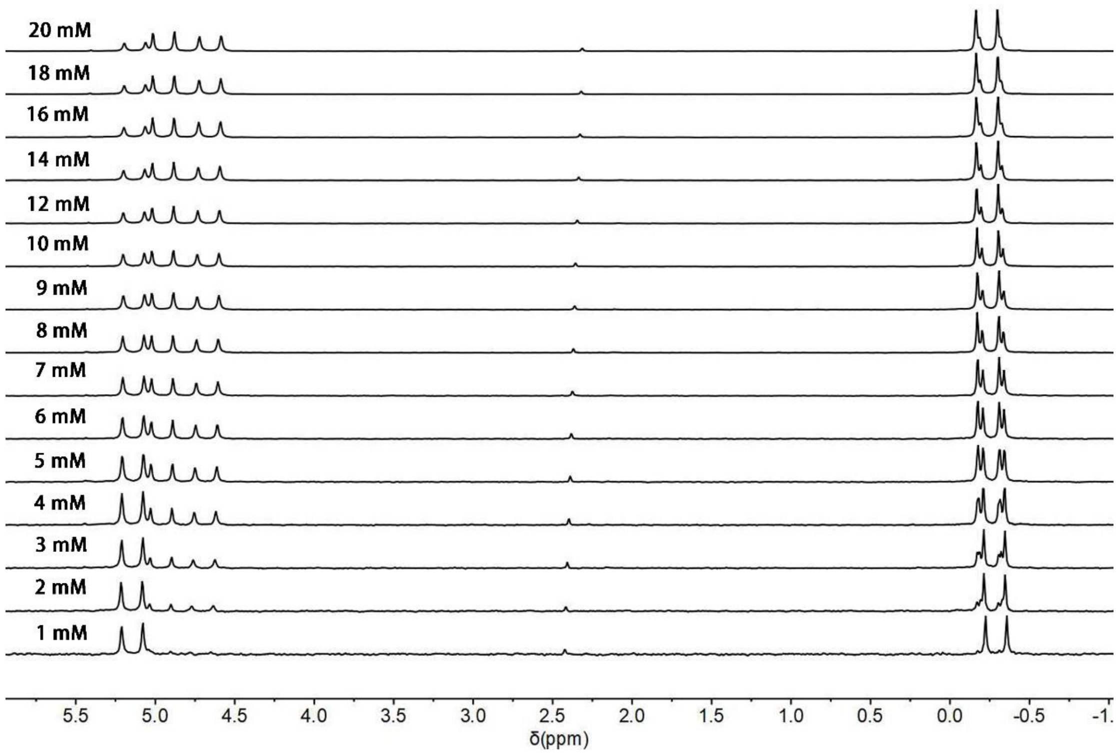

Supplementary Figure 14. $^{31}P\{^{1}H\}$ NMR (160 MHz, acetone-d_6, 298K) spectra of **8** at different concentrations.

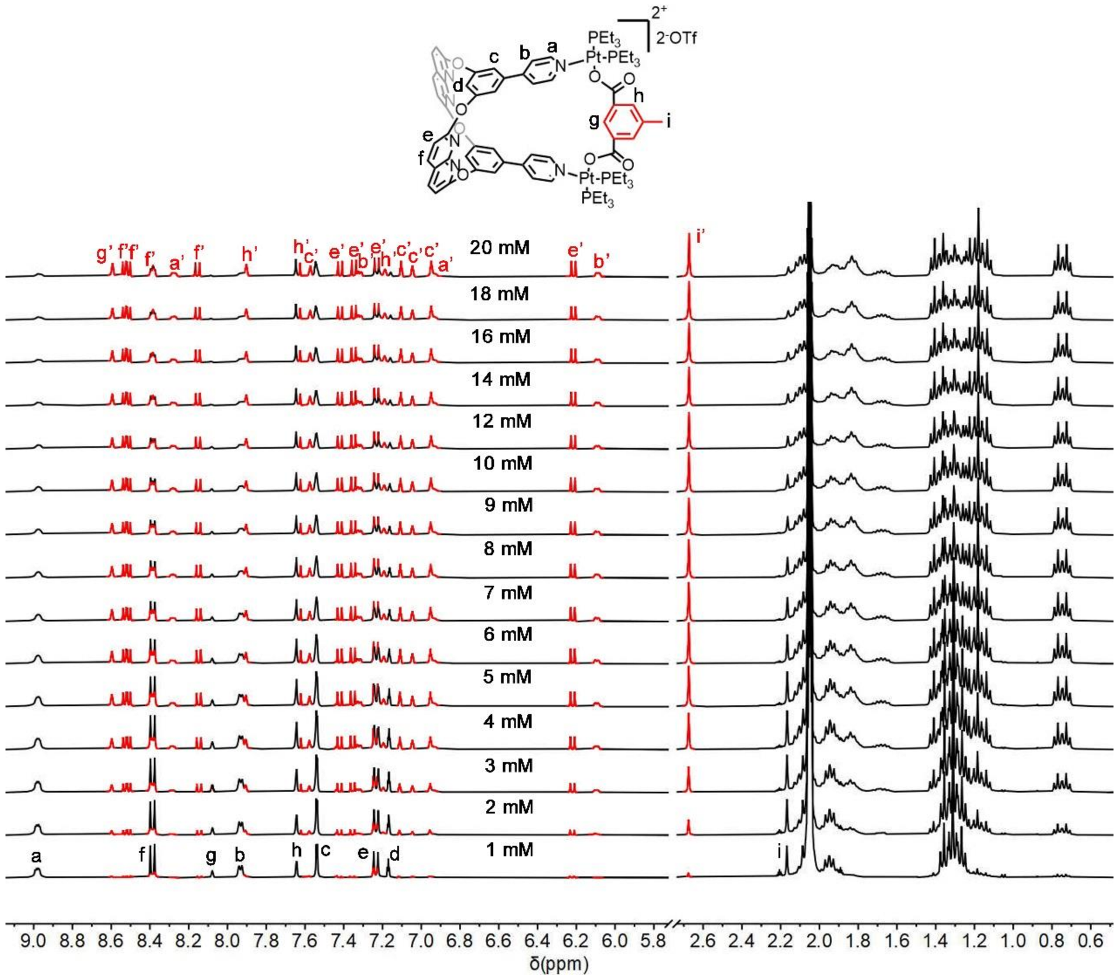

Supplementary Figure 15. ^{1}H NMR (400 MHz, acetone-d_6, 298K) spectra of **8** at different concentrations.

Tables of conversions of [2]catenanes 4+4, 6+6 and 8+8 in different concentrations

Supplementary Table 4. Conversions of **4+4** in different concentration of **4**

Concentration of **4** (mM) in acetone-d_6	Conversions of **4+4** (%)
1	12
2	23
3	35
4	43
5	50
6	54
7	57
8	61
9	63
10	64
12	67
14	70
16	71

18	73
20	74

Supplementary Table 5. Conversions of **6+6** in different concentration of **6**

Concentration of **4** (mM) in acetone-d_6	Conversions of **6+6** (%)
1	14
2	36
3	48
4	55
5	60
6	64
7	67
8	69
9	71
10	74
12	76
14	78
16	80
18	80
20	81

Supplementary Table 6. Conversions of **8+8** in different concentration of **8**

Concentration of **4** (mM) in acetone-d_6	Conversions of **8+8** (%)
1	18
2	31
3	40
4	47
5	51
6	57
7	59
8	61
9	62
10	65
12	70
14	70
16	73
18	74
20	75

2D COSY NMR spectra of 6+6 and 4+4

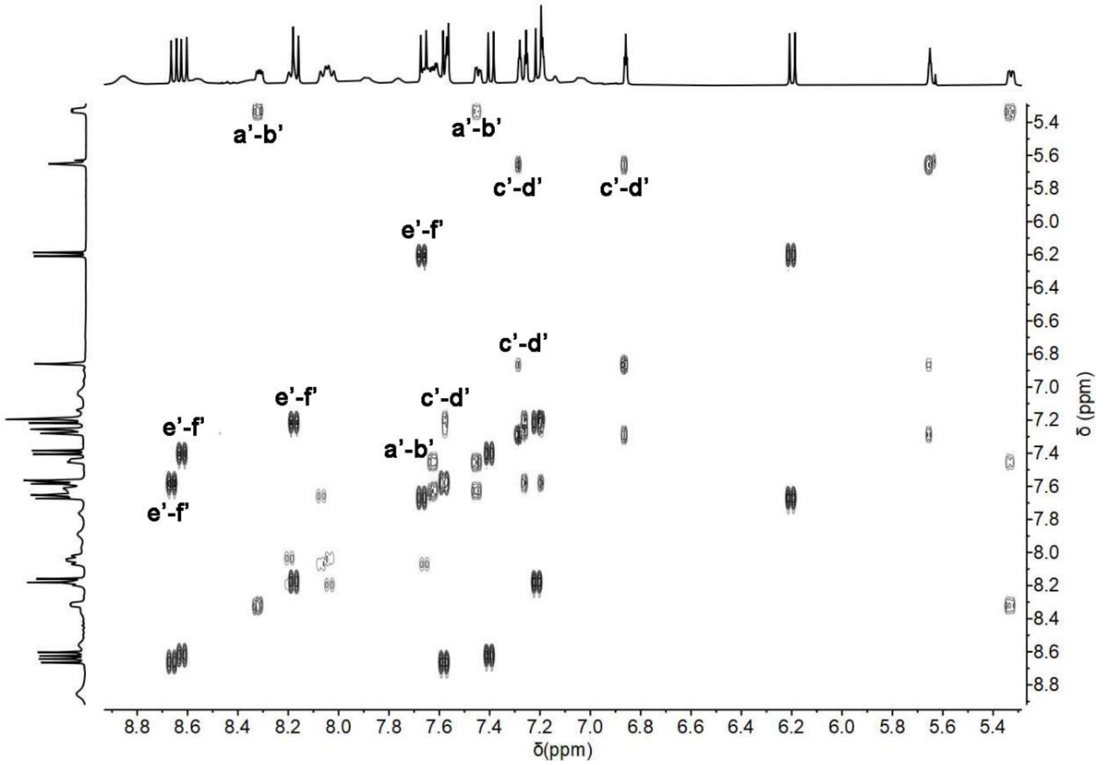

Supplementary Figure 16. ^{1}H-^{1}H COSY NMR spectrum (600 MHz, acetone-d_6, 298 K) of **6** at 20 mM.

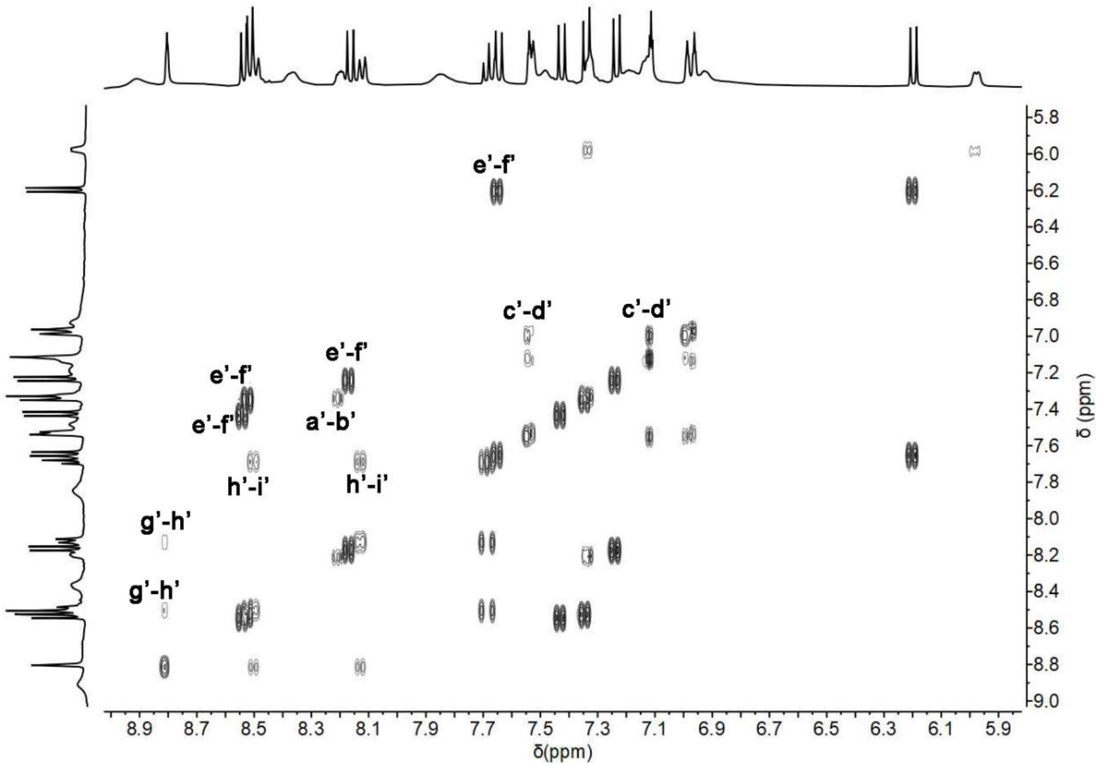

Supplementary Figure 17. ^{1}H-^{1}H COSY NMR spectrum (600 MHz, acetone-d_6, 298 K) of **4** at 20 mM.

Concentration dependent NMR experiments for 4 & 6, 8 & 6 1:1 mixtures

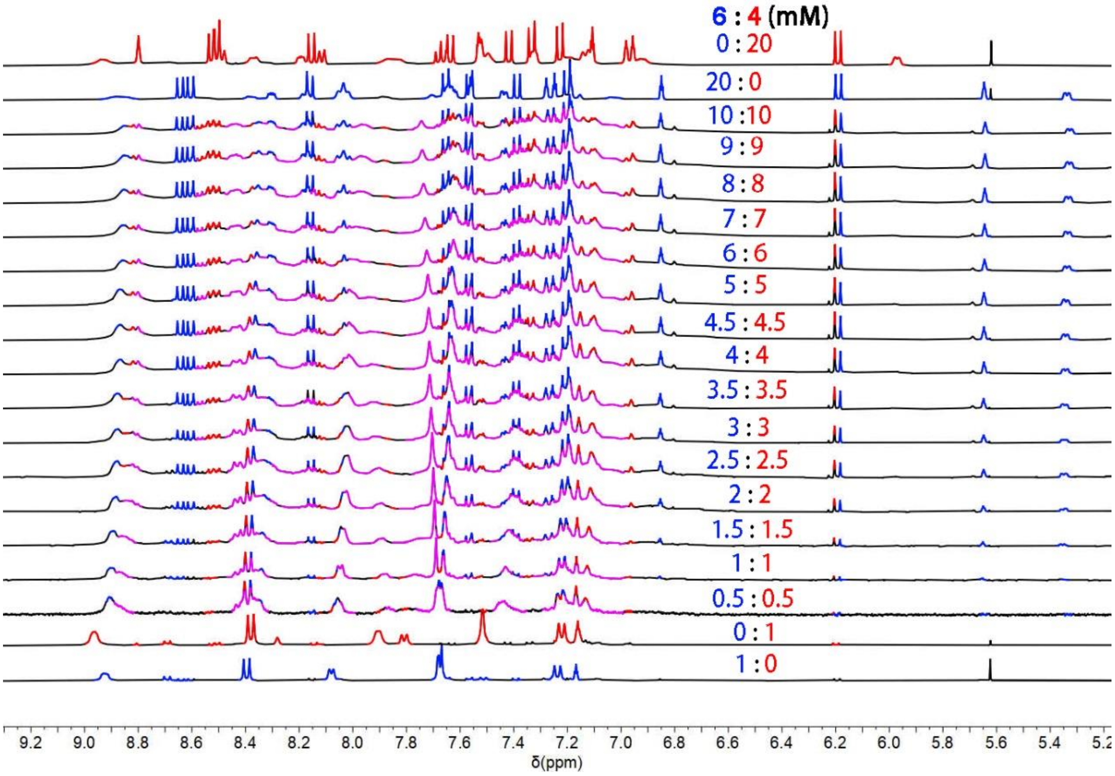

Supplementary Figure 18. ^{1}H NMR (400 MHz, acetone-d_6, 298K) spectra of **4** and **6** mixture at different concentrations.

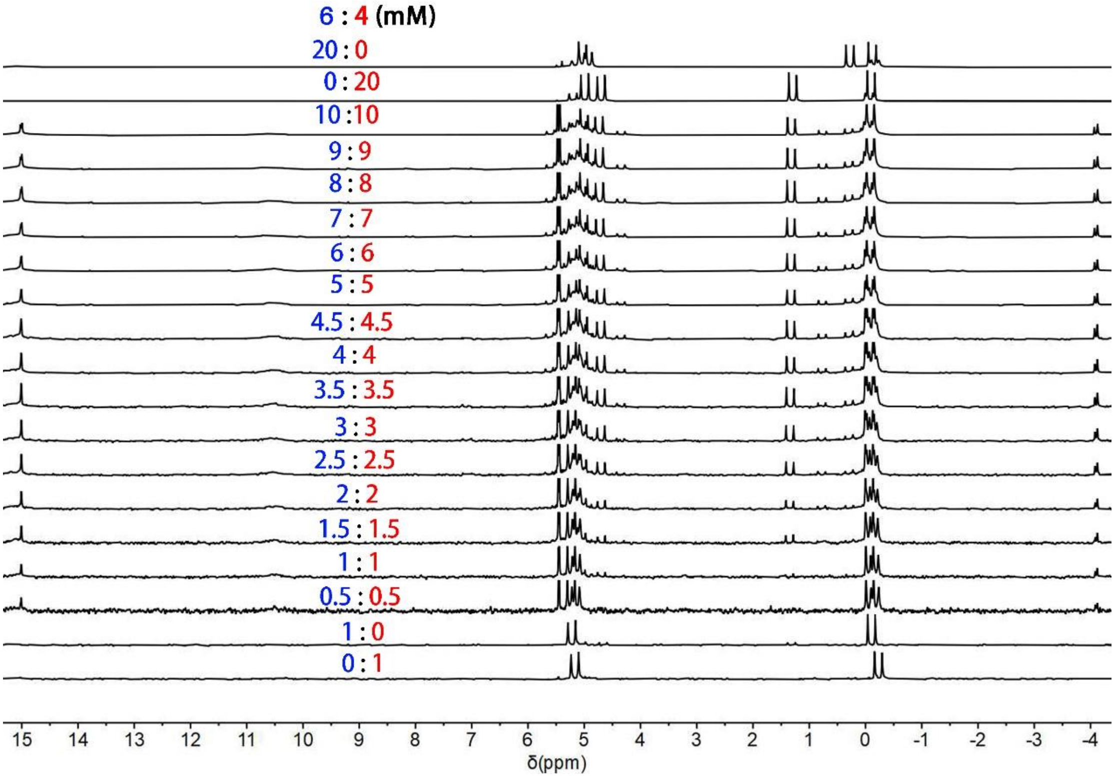

Supplementary Figure 19. ^{31}P{^{1}H} NMR (160 MHz, acetone-d_6, 298K) spectra of **4** and **6** mixture at different concentrations.

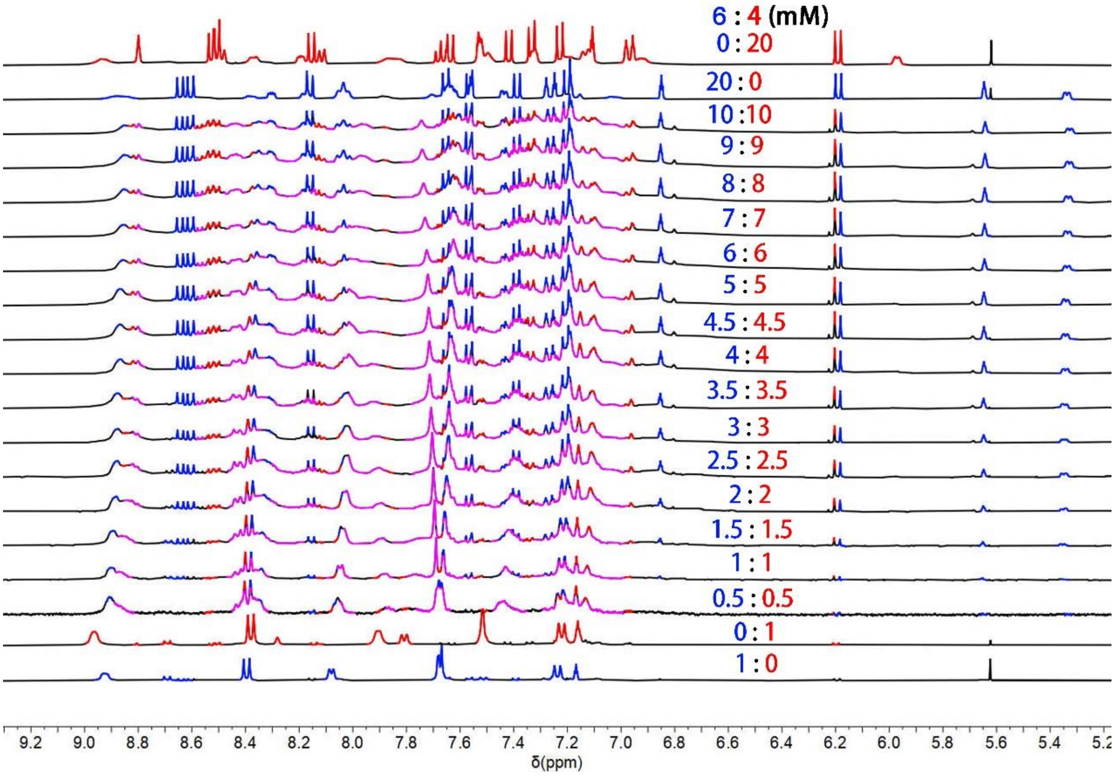

Supplementary Figure 20. ^{1}H NMR (400 MHz, acetone-d_6, 298K) spectra of **6** and **8** mixture at different concentrations.

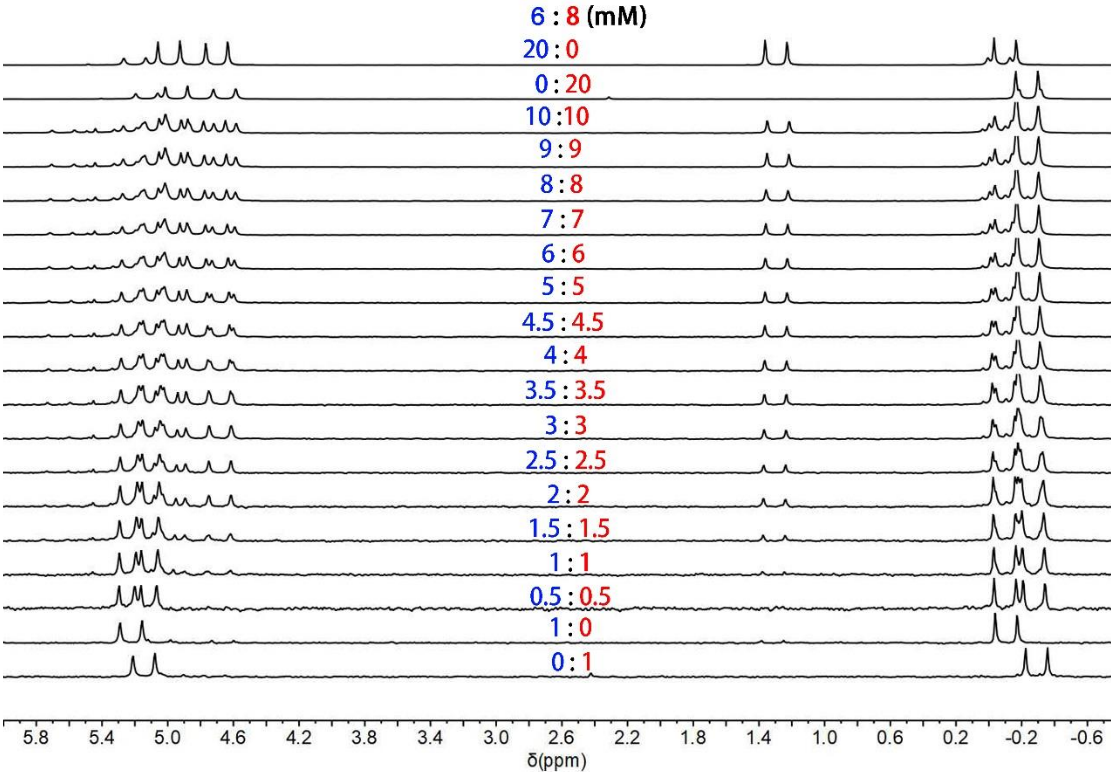

Supplementary Figure 21. $^{31}P\{^{1}H\}$ NMR (160 MHz, acetone-d_6, 298K) spectra of **6** and **8** mixture at different concentrations.

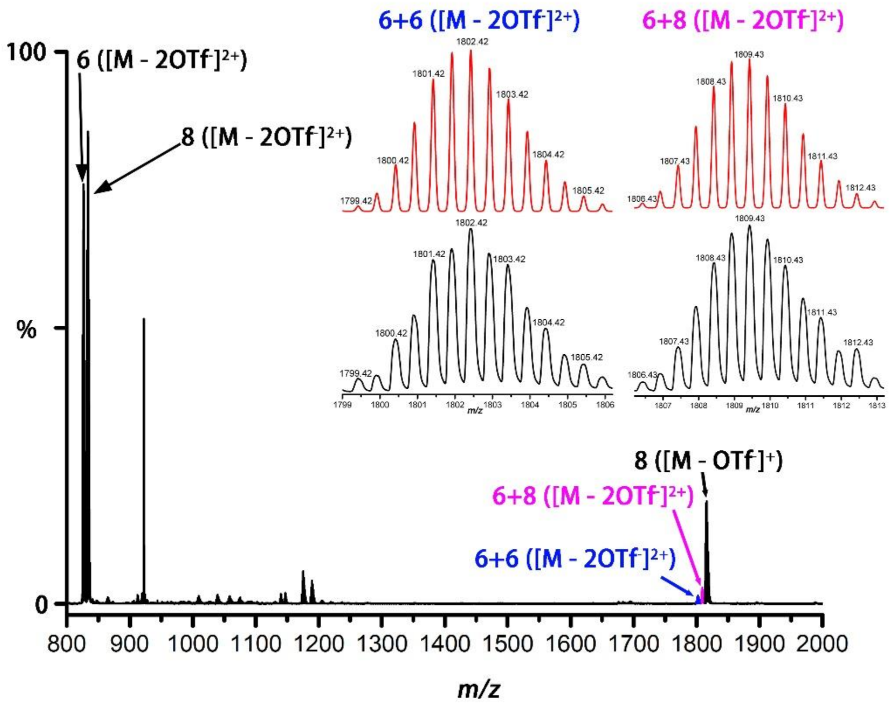

Supplementary Figure 22. Experimental (black) and calculated (red) electrospray ionization mass spectrum of 1:1 mixtrue of **6** & **8**.

Supplementary Table 7. Conversions of **6+8** in different concentration of **6** and **8** 1:1 mixture

Total Concentration of metallacages in acetone-d_6	Conversions of **6+8** (%)
1	-
2	4
3	7
4	9
5	10
6	11
7	12
8	13
9	13
10	14
12	15
14	15
16	15
18	15
20	17

Theoretical and Computational Methods

The global-minimum (GM) structures of [2]catenane **4+4**, **4+6** and **6+6** were searched using TGMin (v.3) program[1,2,3] based on the basin-hopping algorithm interfaced with the GFN2-xTB package[4], respectively. The generalised Born and surface area solvation (GBSA) model[5] with acetone as solvent was also taken into consideraion. In total, 67320 isomers of **4+4**, 54589 isomers of **4+6**, and 55020 isomers of **6+6** were searched, respectively.

Subsequently, the GM structure of [2]catenane **4+4**, **4+6** and **6+6** were re-optimized by using B3LYP exchange-correlation functional[6,7] and def2-TZVP basis sets[8] in Gaussian (version G16RevB.01) program[9]. The Grimme's D3 correction[10] was also applied to take the dispersion correction into account. Furthermore, the universal solvation model based on density (SMD)[11] with acetone as solvent was also applied in order to take the solvent effect into account. The binding energies were calculated following the thermodynamic conventions：BDE = $\sum E_{complex} - \sum(E_{fragment(i)})$.

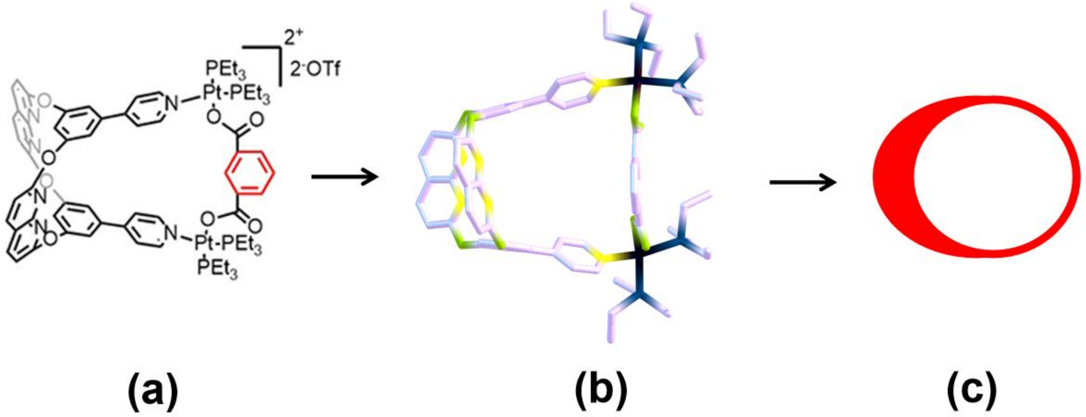

Supplementary Figure 23. (a) the skeleton structure; (b) the geometrical and (c) topological structure of monomer **4** optimized at the B3LYP/def2-TZVP level of theory.

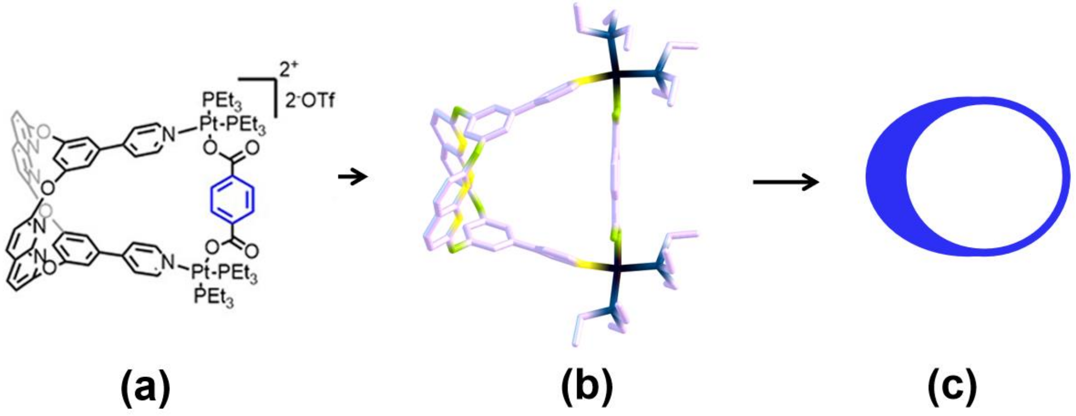

Supplementary Figure 24. (a) The skeleton structure; (b) the geometrical and (c) topological structure of monomer **6** optimized at the B3LYP/def2-TZVP level of theory.

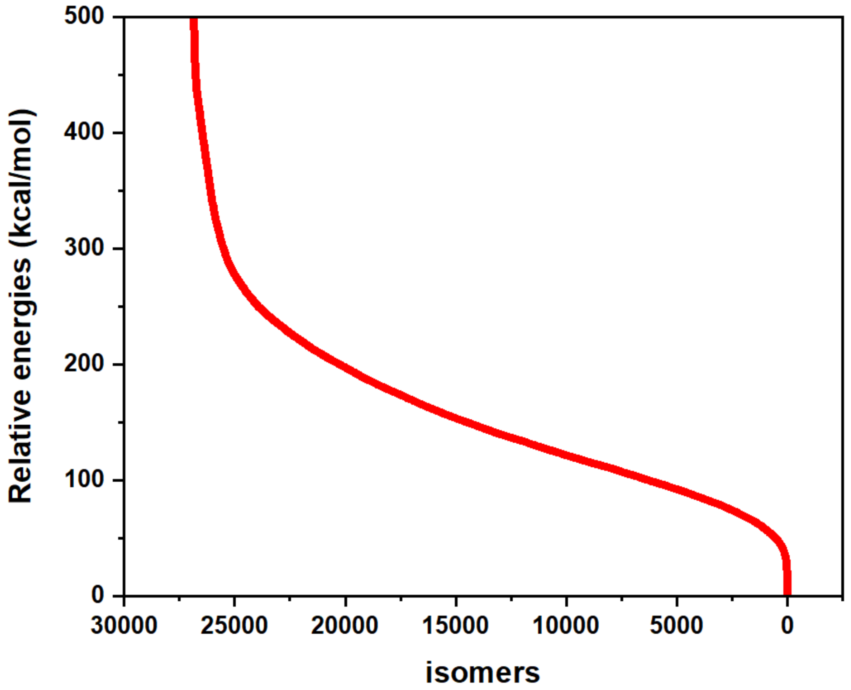

Supplementary Figure 25. Relative energies (in kcal/mol) of various isomers of [2]catenane **4+4** obtained from conformation search by TGMin-3 software at the GFN2-xTB level of theory.

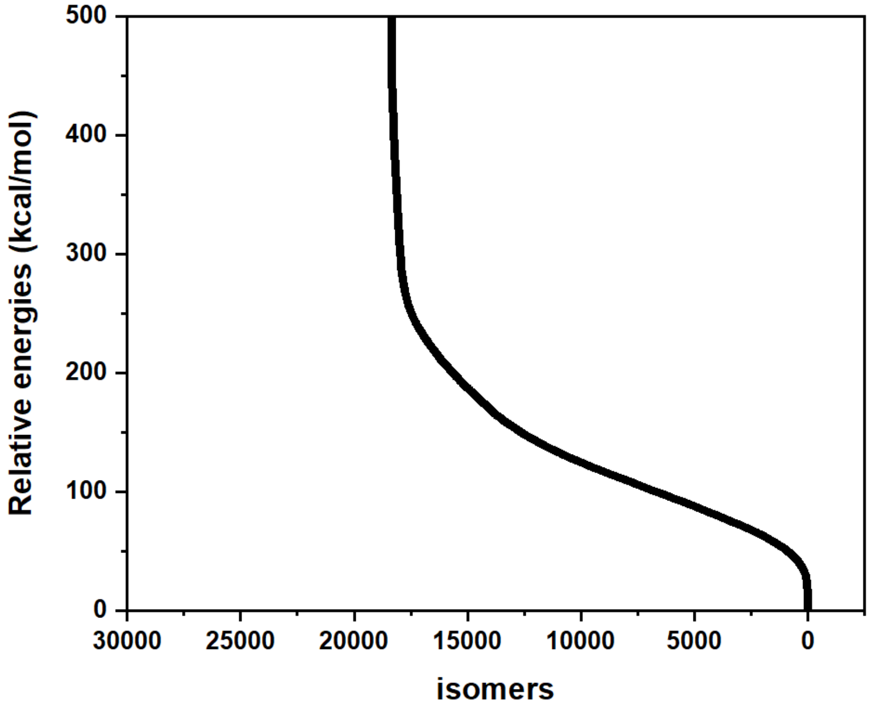

Supplementary Figure 26. Relative energies (in kcal/mol) of various isomers of [2]catenane **4+6** obtained from conformation search by TGMin-3 software at the GFN2-xTB level of theory.

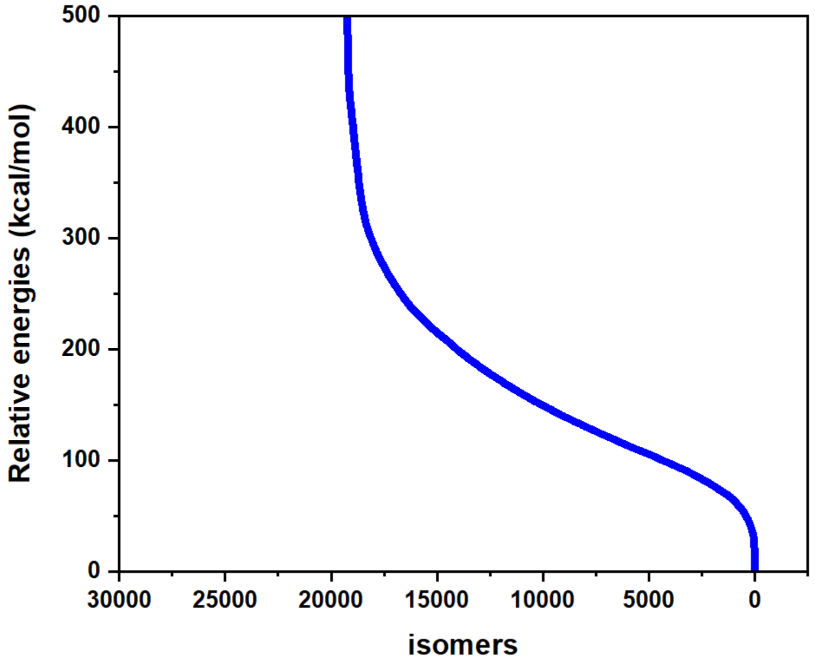

Supplementary Figure 27. Relative energies (in kcal/mol) of various isomers of [2]catenane **6+6** obtained from conformation search by TGMin-3 software at the GFN2-xTB level of theory.

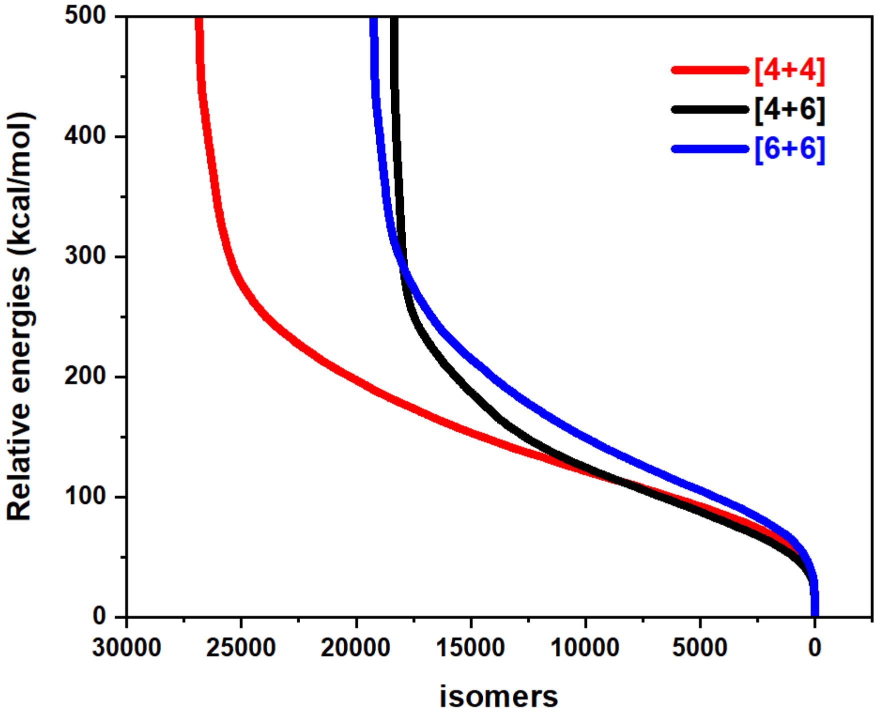

Supplementary Figure 28. Relative energies (in kcal/mol) of various isomers of [2]catenanes **4+4**, **4+6** and **6+6** obtained from conformation search by TGMin-3 software at the GFN2-xTB level of theory, respectively.

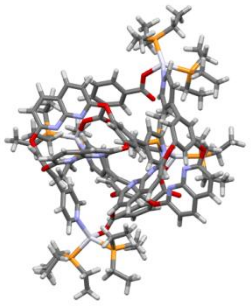
(0.00)

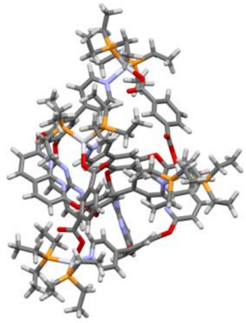
(6.75)

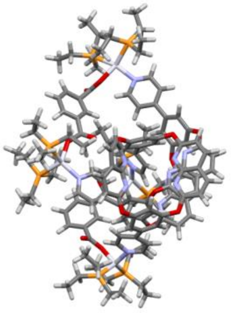
(12.87)

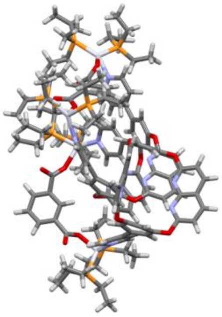
(13.78)

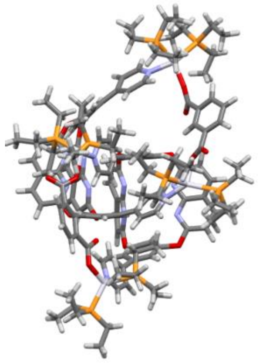
(13.95)

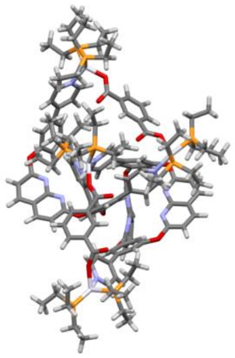
(14.23)

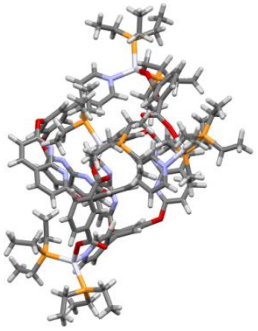
(15.73)

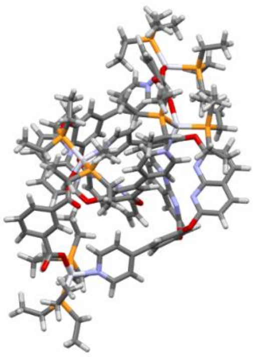
(17.23)

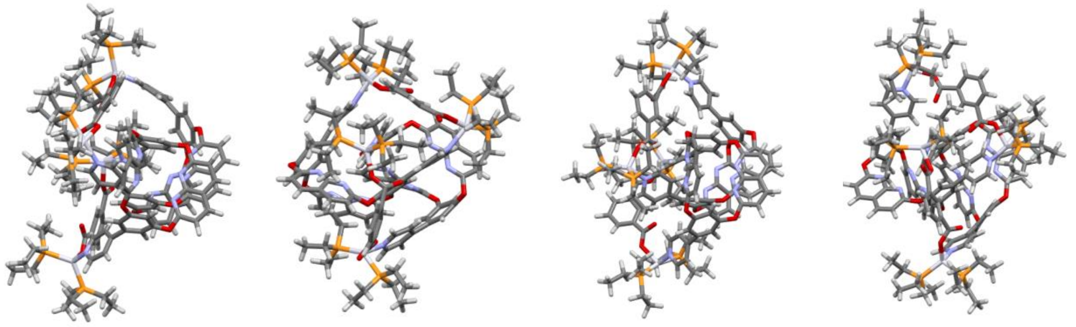

(18.43) (18.90) (19.29) (20.10)

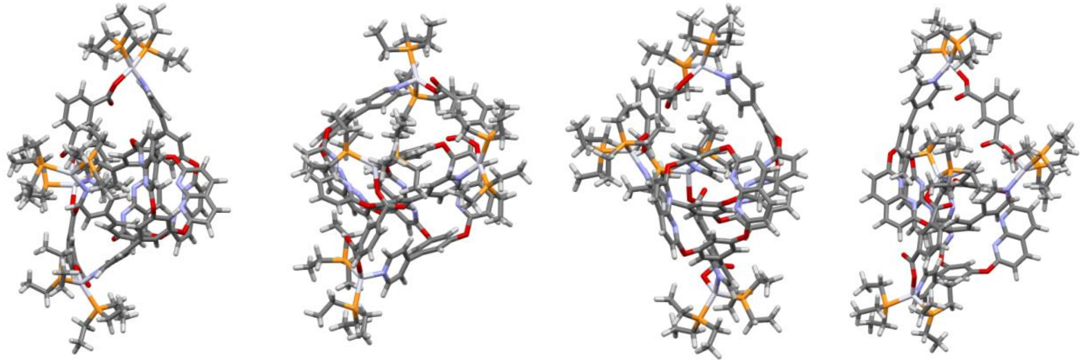

(20.28) (20.66) (21.51) (21.85)

Supplementary Figure 29. Selected geometrical structures and relative energies (in kcal/mol) of [2]catenane **4+4** obtained from conformation search by TGMin-3 software at the GFN2-xTB level of theory.

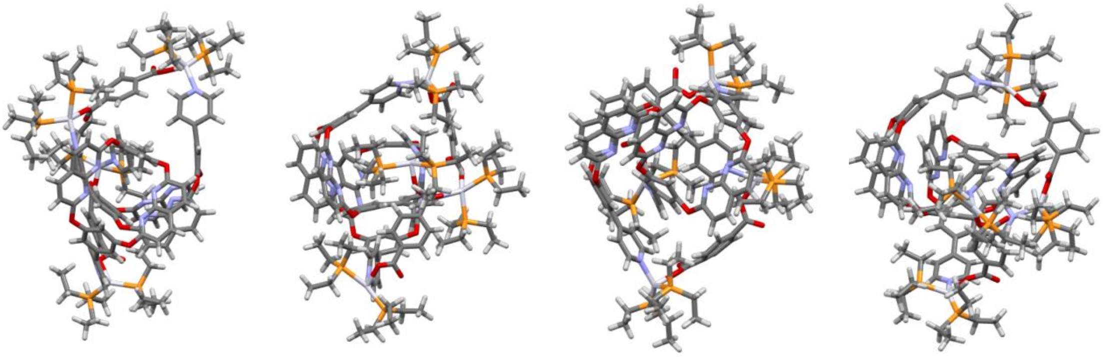

(0.00) (6.40) (8.80) (10.90)

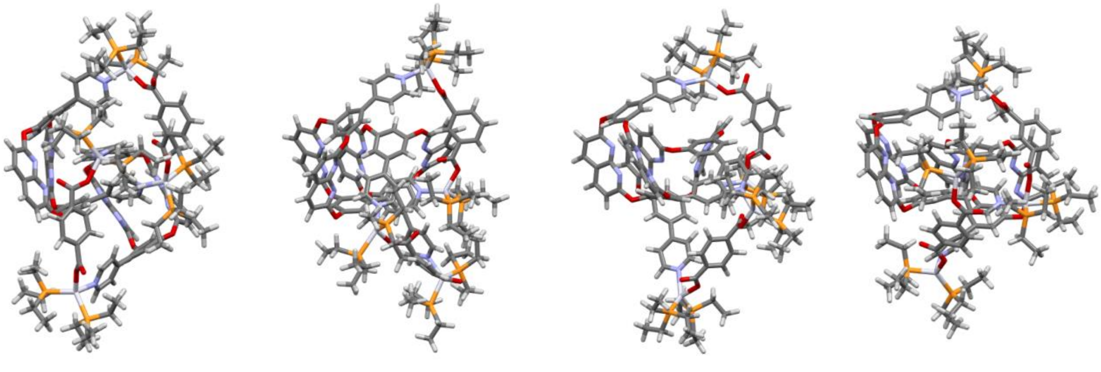

(11.29) (11.43) (12.83) (13.35)

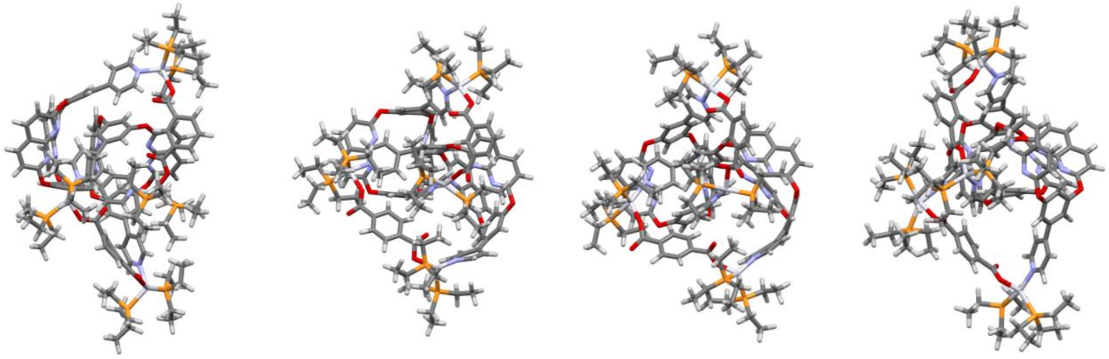

(14.24) (15.24) (15.26) (16.30)

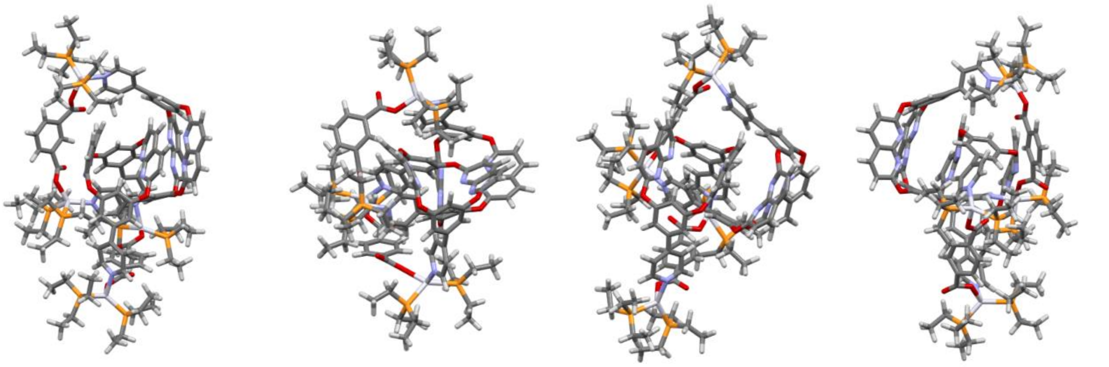

(17.20) (17.72) (18.02) (18.25)

Supplementary Figure 30. Selected geometrical structures and relative energies (in kcal/mol) of [2]catenane **4+6** obtained from conformation search by TGMin-3 software at the GFN2-xTB level of theory.

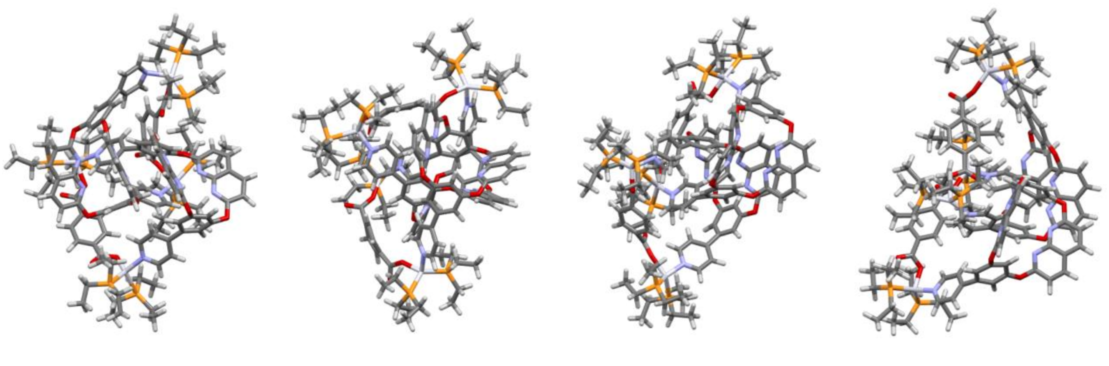

(0.00) (6.11) (8.60) (8.92)

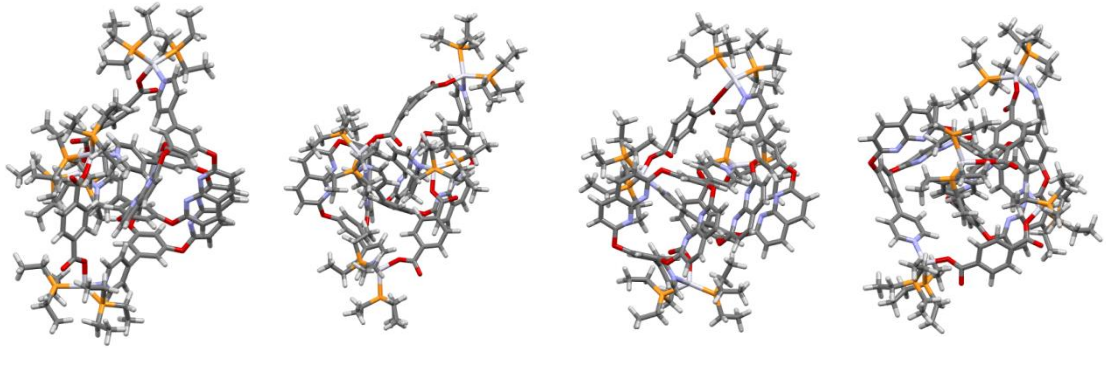

(9.33) (12.83) (16.12) (17.27)

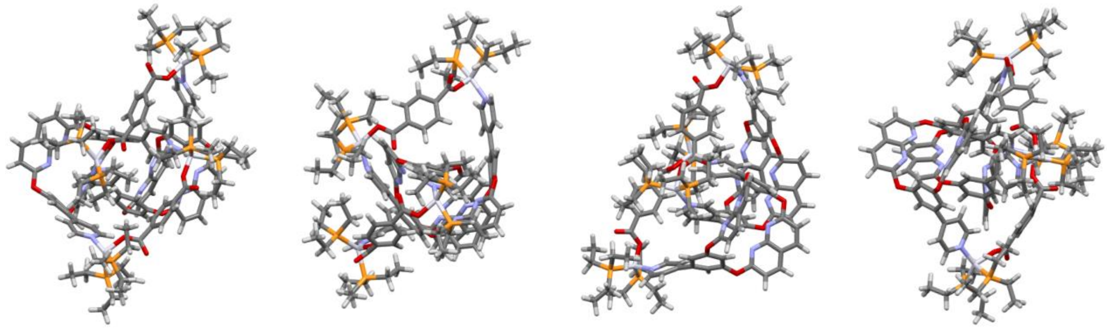

(17.60) (19.57) (19.70) (19.79)

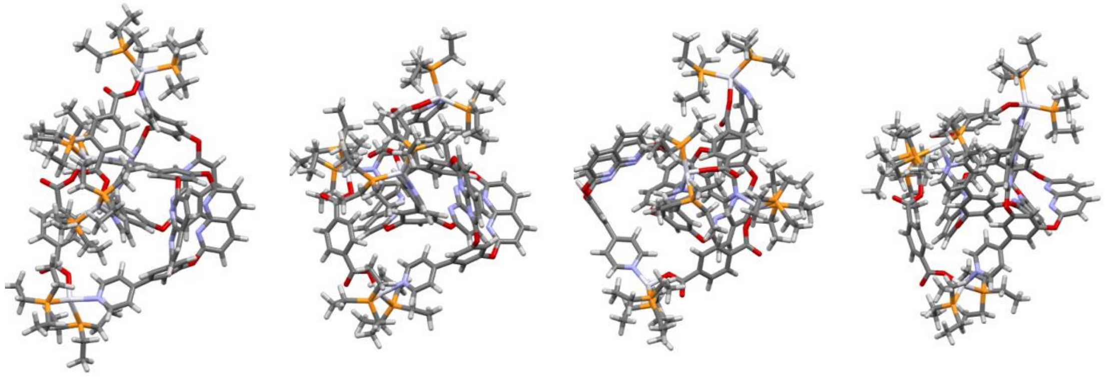

(19.98) (20.74) (21.01) (21.26)

Supplementary Figure 31. Selected geometrical structures and relative energies (in kcal/mol) of [2]catenane **6+6** obtained from conformation search by TGMin-3 software at the GFN2-xTB level of theory.

1 Zhao, Y., Chen, X., Li, J. TGMin: A global-minimum structure search program based on a constrained basin-hopping algorithm. *Nano Res.* **2017**, *10*, (10), 3407-3420.

2 Chen, X.; Zhao, Y.-F.; Wang, L.-S.; Li, J., Recent progresses of global minimum searches of nanoclusters with a constrained Basin-Hopping algorithm in the TGMin program. *Comput. Theor. Chem.* **2017**, *1107*, 57-65.

3 Chen, X.; Zhao, Y. F.; Zhang, Y. Y.; Li, J., TGMin: An efficient global minimum searching program for free and surface-supported clusters. *J. Comput. Chem.* **2019**, *40*, (10), 1105-1112.

4 Bannwarth, C.; Ehlert, S.; Grimme, S., GFN2-xTB-An Accurate and Broadly Parametrized Self-Consistent Tight-Binding Quantum Chemical Method with Multipole Electrostatics and Density-Dependent Dispersion Contributions. *J. Chem. Theory. Comput.* **2019**, *15*, (3), 1652-1671.

5 Kollman, P. A.; Massova, I.; Reyes, C.; Kuhn, B.; Huo, S. H.; Chong, L.; Lee, M.; Lee, T.; Duan, Y.; Wang, W.; Donini, O.; Cieplak, P.; Srinivasan, J.; Case, D. A.; Cheatham, T. E. Calculating structures and free energies of complex molecules: Combining molecular mechanics and continuum models *Acc. Chem. Res.* **2000**, 33 (12), 889-897.

6 Becke, A. D., Density-Functional Exchange-Energy Approximation with Correct Asymptotic Behavior. *Phys Rev A* **1988**, 38, 3098-3100.

7 Lee, C.; Yang, W.; Parr, R. G., Development of the Colle-Salvetti Correlation-Energy Formula into a Functional of the Electron Density. *Phys.*

Rev. B **1988**, 37, 785-789.
8 Weigend, F.; Ahlrichs, R., Balanced Basis Sets of Split Valence, Triple Zeta Valence and Quadruple Zeta Valence Quality for H to Rn: Design and Assessment of Accuracy. *Phys. Chem. Chem. Phys.* **2005**, 7, 3297-3305.
9 Frisch, M.; Trucks, G.; Schlegel, H.; Scuseria, G.; Robb, M.; Cheeseman, J.; Scalmani, G.; Barone, V.; Petersson, G.; Nakatsuji, H., Gaussian 16. In Revision B, Gaussian, Inc., Wallingford CT: **2016**; Vol. 3.
10 Grimme, S.; Antony, J.; Ehrlich, S.; Krieg, H., A Consistent and Accurate Ab Initio Parametrization of Density Functional Dispersion Correction (Dft-D) for the 94 Elements H-Pu. *J. Chem. Phys.* **2010**, 132, 154104.
11 Marenich, Aleksandr V et al. Universal solvation model based on solute electron density and on a continuum model of the solvent defined by the bulk dielectric constant and atomic surface tensions. *J Phys Chem B*. **2009**, 113(18):6378-96.
